# Molecular Modeling to Study Dendrimers for Biomedical Applications

**DOI:** 10.3390/molecules191220424

**Published:** 2014-12-08

**Authors:** Nuno Martinho, Helena Florindo, Liana Silva, Steve Brocchini, Mire Zloh, Teresa Barata

**Affiliations:** 1 Research Institute for Medicines (iMed.ULisboa), Faculty of Pharmacy, Universidade de Lisboa, Av. Professor Gama Pinto, Lisbon 1649-003, Portugal; 2 Department of Pharmaceutics, The School of Pharmacy, University of London, 29/39 Brunswick Square, London WC1N 1AX, UK; 3 Department of Pharmacy, University of Hertfordshire, College Lane, Hatfield AL10 9AB, UK

**Keywords:** dendrimers, molecular dynamics, molecular docking, biological interactions, drug encapsulation, dendrimer-drug interaction, biomaterials, hyperbranched polymer design, molecular recognition, nanomedicine

## Abstract

Molecular modeling techniques provide a powerful tool to study the properties of molecules and their interactions at the molecular level. The use of computational techniques to predict interaction patterns and molecular properties can inform the design of drug delivery systems and therapeutic agents. Dendrimers are hyperbranched macromolecular structures that comprise repetitive building blocks and have defined architecture and functionality. Their unique structural features can be exploited to design novel carriers for both therapeutic and diagnostic agents. Many studies have been performed to iteratively optimise the properties of dendrimers in solution as well as their interaction with drugs, nucleic acids, proteins and lipid membranes. Key features including dendrimer size and surface have been revealed that can be modified to increase their performance as drug carriers. Computational studies have supported experimental work by providing valuable insights about dendrimer structure and possible molecular interactions at the molecular level. The progress in computational simulation techniques and models provides a basis to improve our ability to better predict and understand the biological activities and interactions of dendrimers. This review will focus on the use of molecular modeling tools for the study and design of dendrimers, with particular emphasis on the efforts that have been made to improve the efficacy of this class of molecules in biomedical applications.

## 1. Introduction

Dendrimer is a name derived from the Greek word *dendros* (tree or branch) and *meros* (part of) [1] to describe a class of macromolecular hyperbranched polymers with a well-defined radial branching architecture. Contrary to most other polymers—*i.e.*, linear and branched polymers—dendrimers often do not rely on statistical description of average structure and molecular weight characteristics [2]. Dendrimers can be synthesised by iterative stepwise reactions of monomers. The synthetic route is often characterized as either divergent (from the centre outwards) or convergent (from the outside towards the centre) [3].

**Figure 1:**
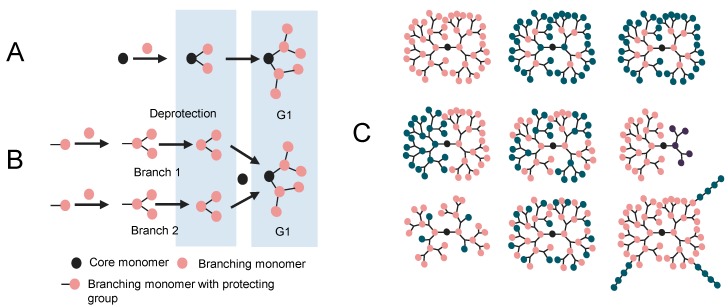
Structural properties of dendrimers; (**A**) Divergent synthesis; (**B**) Convergent synthesis; (**C**) Different topological structures that can be obtained from synthesis.

Of these two methods, divergent strategies appear to be better suited for large-scale production. The synthesis starts from the core where consecutive generations or layers, of what is often a tri-functional monomer are added. Moreover, often two functional groups of this monomer are masked (*i.e.*, protected from reaction). Each monomer then undergoes reaction at the one available functional moiety leaving the two masked moieties available for repeat reaction after deprotection. Hence each iterative reaction sequence or generation doubles the number of end groups as the dendrimer grows (Figure 1A). This method, however, has some drawbacks since incomplete or side reactions results in structural heterogeneity, especially as the number of generations increases. This is often thought to be due to increased steric interactions as the number of end groups increase. There are, however, purification challenges (see [4,5]) with this strategy, when trying to separate dendrimers that differ by small differences in monomer incorporation. To overcome these hurdles, at small scale, convergent methods of synthesis were developed [6]. In these methods the structure is built from the ends of the branches towards the dendrimer core. The monomers undergo reactions to form the arms that are then covalently linked to the core (Figure 1B). Convergent methods, though solving the purification and side reaction problems of the divergent methods do not allow the synthesis of high generation dendrimers due to steric effects that are encountered when trying to bring the dendrimer fragments (called dendrons) together to react with a reagent that forms the core [5].

Generally, dendrimer structure can be described by the: (i) core; (ii) branches; (iii) terminal groups and (iv) void space. Due to the iterative chemistry underlying dendrimer synthesis, these macromolecules are usually described by their generation number, for example generation 0 (G0), 1 (G1), 2 (G2) and so on. It is also common to describe the dendrimers as half-generation when the terminal groups of the last monomer are altered (e.g., G1.5 or G1.5-COOH) to change chemical functionality of the end groups.

As with all molecules, dendrimer structure correlates with properties and applications. There are key intrinsic features that include the potential for the precise control of molecular weight, branching and interior chemistry. Interestingly, since dendrimers are hyperbranched they tend to have much lower viscosity properties compared to linear polymers of comparable molecular weight. For some dendrimers, the intrinsic viscosity reaches a maximum with the increase in molecular weight and then decreases with further increase of molecular weight (see [7,8]). Synthetic versatility can usually be achieved to give multivalent terminal groups and a wide variety of cores (see [9,10] and as reviewed elsewhere [1,11,12,13]). A vast array of dendrimer structures have been prepared since dendrimers were first described [14] and the potential number of structures of these molecules is almost limitless (Figure 1C).

In principle, dendrimers can be composed of any type of monomer and hence different physicochemical behaviours can be expected based on the kind of monomers used. A widely described example is the poly(amidoamine) (PAMAM) dendrimers. These are the most extensively studied dendrimers in the biomedical field. Other commonly studied dendrimers include poly(propylene imine) (PPI or DAB) [15], polypeptides [16], poly(ester) [17], triazines [18] and phosphorus-containing [19] dendrimers. The final end groups can be further modified with other chemical moieties including recognition moieties (e.g., folate, glutathione, RGD, immunoglobulins (Ig)), lipids and bioresponsive elements or polymers (e.g., PEG (polyethyleneglycol)) to optimise the biological properties [20,21,22].

Dendrimers can also act as materials, excipients or active molecules and can be applied as catalysts, in the preparation of hydrogels, biosensors (molecular recognition), tissue engineering, drug delivery systems, transfection agents and even as therapeutic agents or biomimetics. Hence the recurrent need is to develop a better understanding to aid the design and increase the performance of these macromolecules (for further reading see [23,24,25,26,27,28,29,30]).

Computer simulation techniques are a valuable tool to predict the properties of dendrimers at the molecular level. By gaining insight into the key biomedical factors that correlate with dendrimer structure one can design and optimize their properties for various applications. Nevertheless there is still a great challenge to develop novel dendrimers as any modification (e.g., terminal groups) in the dendrimer structure is likely to alter its morphological properties and hence its biological activity and toxicity (reviewed elsewhere [31,32,33]). Therefore a better understanding how these macromolecules interact with the biological systems is required to develop safer therapeutics.

Although the use of molecular modeling techniques for dendrimers can be a powerful tool, there is a lack of dedicated software for these macromolecules. In particular, force field development still presents a significant challenge to model dendrimer properties. Herein we review the current strategies used to computationally model dendrimers and describe how these models have been applied to develop dendrimers for use in drug delivery and as therapeutic agents.

## 2. Molecular Modeling of Dendrimers

**Figure 2:**
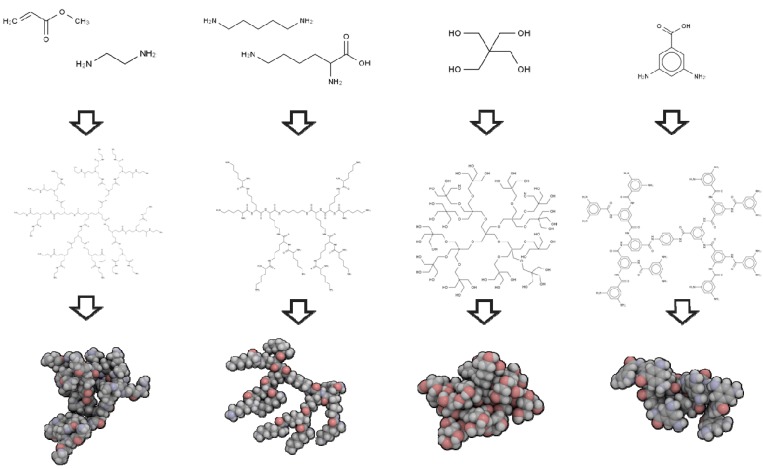
Topological structures obtained with different core and branching monomers; From top to bottom: monomeric units, 2D structure of a G2 dendrimer, 3D structure of G2 dendrimer.

Several parameters impact on the architecture of dendrimers in aqueous medium. These include generation number (size), type of monomer (e.g., spacer length, density groups, void space and branching units) and surface terminal groups (charge and hydrophobicity) (see Figure 2). Valence of counter-ions in the solution and presence of other molecules, pH or ion salt concentration, also play a role. Several experimental techniques are used to probe dendrimer structure, including nuclear magnetic resonance (NMR), mass spectroscopy, infrared and Raman spectroscopy, fluorescence and small angle neutron scattering (SANS) (as reviewed in [34]). Although these techniques provide valuable information about the size and molecular constituents they have some limitations. For example, the determination of spatial configuration and geometric characterization [35] can be challenging. Since dendrimers are essentially a repetitive sequence of monomers, probing configurations is limited due to the low differentiation of the signal. Furthermore, flexible dendrimers can have a number of permissible configurations in solution with a rapid interchange between them [35,36,37,38]. Nevertheless, sometimes, specific chemical groups can be used to probe their local environment if they are different enough. As an example, amide protons in a poly(l-glutamic acid) dendrimers had separate NMR chemical shifts and those were used to probe their exposure to solvent in two different generations by changing the temperature [39], as well as to obtain information about flexibility and the association of lipidic peptide G3 dendrons [40].

Computational techniques can offer valuable insight into the study and exploration of the properties of complex systems such as dendrimers. Properties such as conformational analysis, molecular interaction (biological and drugs) and validation of experimental data can be determined by molecular modeling strategies. Ultimately, molecular modeling strategies have the potential to provide valuable data, which can help to minimize often laborious and expensive laboratory experiments. We believe that experimentation can be made much more efficient when guided by rational design.

Using only theoretical models or even experimental data, simulations can render mechanisms of biological interactions at atomic-level resolution. From the initial theoretical models to complex molecular simulations [41] there has been a great investment in understanding how these macromolecules behave (Section 3). A major benefit from molecular modeling is that allows the user to control every key parameter (e.g., ions, pH, dendrimers’ structure) that might be involved in their biological activity [42,43,44,45]. This comprises simulations in conditions that would be too difficult to study experimentally. Examples of such value could be the study of dendrimers with lack of one dendron or defective parts of the branches. This opens a promising avenue for interpretation and validation of experimental data [46] as well as the design and characterization of many biological interactions.

**Figure 3:**
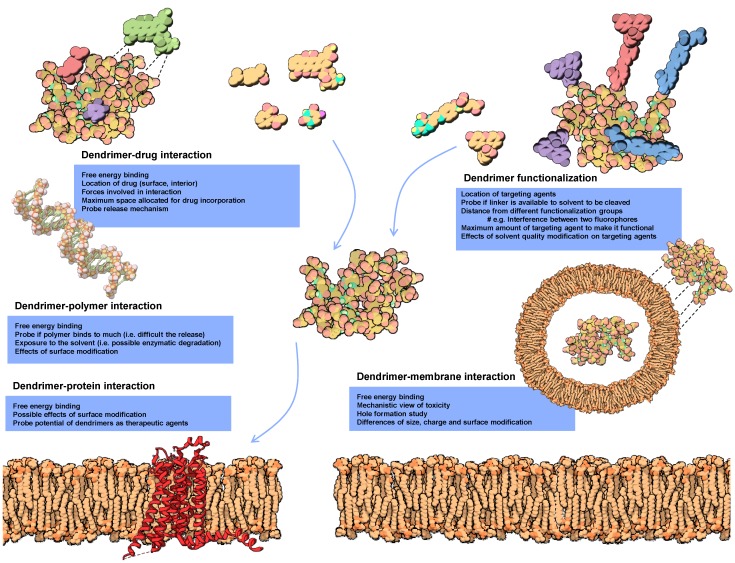
Biomedical applications where MD has been important to probe biological interactions.

Understanding molecular interactions is fundamental to improve biological activity. Based on the energy potential function, molecular dynamic simulations of dendrimers have focused on their behaviour in aqueous solutions (Section 3.1) as well as in interacting with drugs (Section 3.4) and biomolecules including lipid membranes (Section 3.3), nucleic acids and proteins [47,48,49,50,51,52,53] (see Figure 3 and Section 3). Moreover, dendrimers can be seen as protein-like, a sequence of monomers in a three dimensional assembly. Therefore not only their structure can be generated in similar fashion as proteins but modeling techniques such as molecular docking studies can be applied [38,54]. Docking studies allow greater insight on interaction of dendrimers with drugs and biological target. In this regard, docking of several hydrophobic molecules (resveratrol, curcumin and genistein) was able to show that the interaction was mainly in the hydrophobic parts of the dendrimer with some hydrophilic interaction via the hydroxyl groups [55]. Docking studies also contribute to improve dendrimer design, allowing the selection of potential groups that will increase affinity [56,57]. In a recent study, PAMAM dendrimers partially glycosylated were found to dock with MD-2 protein in such a way that was able to prevent its interaction with LPS (Lipopolysaccharide) [52].

The crucial step when using molecular simulations techniques is to establish the main questions and goals for the intended study. With current access to computational power it is possible to perform more and more complex simulations, despite the yet great challenge to accurately describe complex systems, in a reasonable time scale [58]. The prediction of dendrimer properties can be studied under different ensemble configurations (temperature, pressure and volume), which can sometimes be challenging due to the high molecular density and conformations that need to be defined. Depending on the time-scale and properties to be evaluated, there is no single method available that will provide all the different types of information desired. Dendrimers can be simulated alone or in a certain concentration in the solvent, in the presence of small drugs, proteins and nucleic acids or even in the presence of a lipid bilayer. These involve different scales of the number of atoms necessary to simulate and hence the computational cost differs. Ideally, these simulations would be carried with all atoms taken into account (all-atom simulations) to get a finer description of the interactions involved (e.g., hydrogen bonds). However, due to the resources available and the time scale necessary to study certain events (e.g., incorporation of a drug inside the dendrimer), it is sometimes required the use of simplified models. In this regard, coarse-grained simulations have been a valuable tool in particular to study the interactions with lipid bilayers [53,59,60,61].

### 2.1. 3D Structure Generation

To provide a more general modeling procedure to study dendrimers there is a need for an appropriate nomenclature capable of accurately describing their topology and structure. To start with, the naming and description of the 2D structure (topology) of these molecules is still not universally accepted. IUPAC nomenclature is generally an acceptable way of naming molecules and should enable the exact description of any type of a molecule. Though this nomenclature can be applied to dendrimer-like molecules, it becomes less clear with the increase of dendrimers’ size and does not capture all the necessary structural features (e.g., distribution of residues). Nodal nomenclature based on graph theory is also able to describe dendrimers but has not been widely used [62,63].

**Figure 4:**
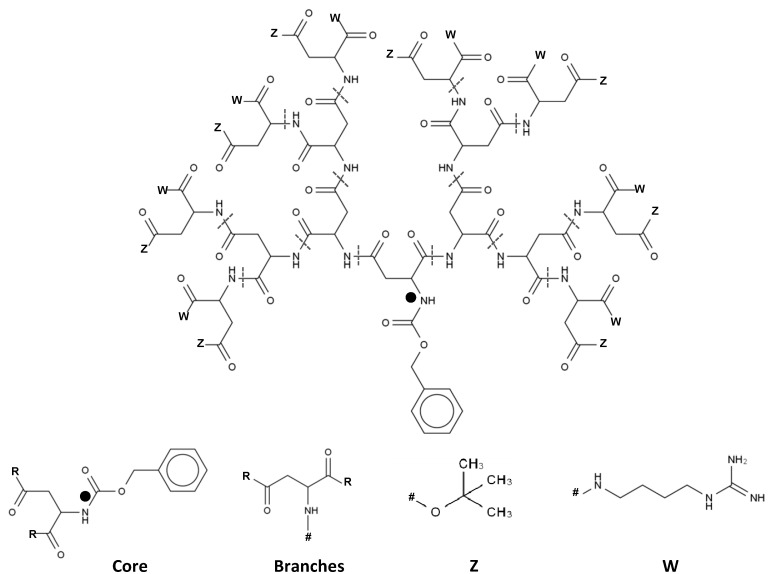
Example of the dotted cap notation for a poly(aspartic acid) dendrimer; The dendrimer is represented by the core, branches and capping groups; This type of notation is useful since several capping groups with different branching points can be easily compared.

Other two nomenclatures for dendrimeric structures are the Newkome-nomenclature [64] and cascadane [65]. Both of these nomenclatures are capable of representing the hyper-branched nature of dendrimers. Both systems make use of the repetitive units that constitute dendrimers to simplify their notation [64,65]. However, as the dendrimers increase in size, the notation becomes complex and difficult to interpret. Further simplification on dendrimer nomenclature, taking advantage of their repetitive topology and symmetry has been proposed with a dotted cap notation [63]. This notation represents dendrimers as building blocks, with a core unit, monomers and capping groups. The core is bound to the monomers which forms the dendrimer framework where the caps are attached [63]. The dotted cap notation then represents the surface of the dendrimer by means of sequential caps (Figure 4). Figure 4 shows an example of how the dotted cap notation would be used to interpret a poly(lysine) dendrimer.

In this case the final notation would be WZ●●WZ●●●WZ●●WZ●●●WZ●●WZ●●●WZ●●WZ or (WZ)_8_ in condensed notation [63]. Here the ● represents the topology distance between the capping groups from the primary atom represented in the core. However, as seen in the example, no information regarding the core or branching units is given, restricting the value of this nomenclature to comparison purposes between complex structures, with variable surface topology. Despite the importance of nomenclature and its contribution to the description of dendrimer topology, it does not provide information on the 3D structure arrangement. Different strategies had to be developed to quickly and effectively generate 3D structures and describe topology. In principle, any chemical drawing software should be sufficient to construct a representation of the dendrimer. Unfortunately the manual assembly of these macromolecules is tedious and highly prone to mistakes, especially with higher generation dendrimers.

To ease this task there are four main packages dedicated to sequential assembly of molecules namely Gromacs [66] and XPLOR [67,68], Starmaker (part of Silico) [69] and Dendrimer Building Toolkit (utilizing AMBERTOOLS) [70]. The first has primarily been used for the dynamic simulation of proteins. XPLOR is used for structure generation based on NMR and X-ray experimental data while the last two packages are dedicated to dendrimer assembly. Regardless of the software package, the topology and parameters of initial monomers have to be defined for the force field that is to be used. All of the information regarding each individual atom and how they are brought together first in monomers and further along as a molecule then needs to be described. It is important to note that the topology and parameter files are specific to each force field. These files often limit the ability to inter-convert between these four molecular assembly packages.

**Figure 5:**
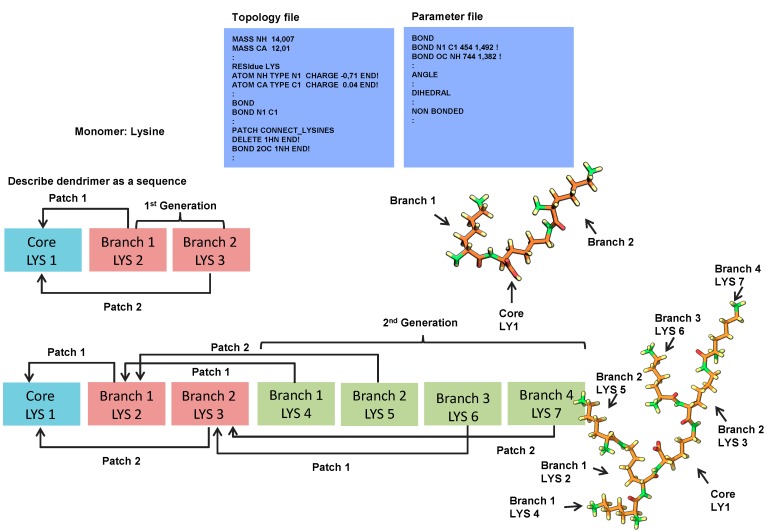
Strategy employed for building dendrimers of different types and generations using XPLOR [71].

Recently, a protocol has been described that facilitates dendrimer construction by describing the dendrimer and the connection between monomers in a sequence [71] using the XPLOR-NIH program (see Figure 5). This requires that the monomers are constructed in the initial stages, which can be achieved by common programs for molecular drawing. These monomers have to be defined within both the topology and parameter files in the XPLOR format. All the information regarding each individual atom and how they are brought together first in monomers and further along as a bigger molecule is described for use in standard molecular dynamics methods. The software then takes the sequence and its connectivity, and assembles each monomer through a simulated annealing protocol so that it can minimize clashes in the structure.

### 2.2. Simulation of Dendrimers

After obtaining the Cartesian (x, y, z) or internal coordinates of either the whole dendrimer or the monomers, the structures are then minimized. Since dendrimers have a large number of atoms, establishing the initial conditions to perform the simulations is a laborious task due to steric overlaps and biased local minima. Moreover, appropriate stereochemistry should be checked as this can lead to erroneous initial structures that are not corrected during the simulation.

Currently there are various approaches to perform molecular simulations. They are based on quantum and molecular mechanics and molecular dynamics. However, in the case of dendrimers, molecular mechanics and dynamics are mostly used due to the high computational cost of quantum calculations. The quantum mechanics or *ab-initio* approach is limited to the lowest generation dendrimers or to defined monomers in an initial conformation. To overcome issues with the size of dendrimers, strategies similar to those used for modeling of proteins such as molecular mechanics methods with reliable parameterization, semi-empirical methods and hybrid quantum/molecular mechanics may give reliable results [50].

The force field (FF) establishes the forces that will be applied to simulate the behaviour of atoms within the dendrimer structure as a function of time [42]. The force fields represent the potential energy and define the forces applied to the system (ensemble of N atoms), which includes the sum of bonded (bond-length, bond-angle, torsion terms) and non-bonded (electrostatic and van der Waals interactions). Commonly force fields that have been used for dendrimers include the AMBER [43,72], CHARMM [73,74,75], GROMOS [38,76], MARTINI [77,78,79] CVFF [80,81], OPLS [29,82,83] and DREIDING Force [84,85]. An overview of the different force fields applied to dendrimers can be seen in Table 1. Force fields use different methods for parameterization and can refer to general atom types or to specific classes of molecules. It is, therefore, extremely important to make sure that the parameters are suitable for the system, when choosing the FF to use, as its choice can lead to different outcomes.

All-atom simulations were conducted with PAMAM dendrimers from G2 to G6 using different force fields (DREIDING, COMPASS and CVFF) and coarse-grained models using the MARTINI [79]. To compare the performance of each FF the radius of gyration (Rg) was measured and compared to experimental values of SAXS (small angles X-ray scattering) [86]. All Rg values for the different all-atom FF were lower when compared to the Rg values determined by SAXS. Similar values were obtained for COMPASS and CVFF force fields with closer values to the experimental results.

DREIDING FF was the least reliable for smaller size dendrimers (G2–G4) and similar to the others for higher generations (G5 and G6). However, the scaling of size in the case of DREIDING FF was found to be similar to previous Brownian dynamic simulations (Rg ~ N^1/3^) and the fractal dimensions (space occupied) were similar to those determined experimentally. For these reasons the authors concluded that DREIDING was the best for determining the Rg for PAMAM dendrimers.

**Table 1:** Examples of software used to build and simulate different types of dendrimers; * NA: the software was not disclosed; ^§^ Free use; ^ƚ^ Commercial use.

Dendrimer Type	Software for Dendrimer Construct	Software Used for Simulations	Force Field	Aim of the Study
Conformational analysis				
PAMAM G2 to G6 [87]	Insight II	Insight II ^ƚ^ CHARMM ^ƚ^	CVFF CHARMM	Structural features at different pH and generations
PAMAM G5 [88]	NA*	MPsim	DREIDING	Effect of pH to study the water dynamics on dendrimers
PAMAM G50% and 90% acetylated [89]	Insight II	AMBER8 ^ƚ^	GAFF	Effect of acetylation on structural features
Glycosylated PAMAM G3.5 [90]	XPLOR	Desmond ^§^	OPLS_2005	Effect of glycosylation on structural features
Triazine G3 and G5 with DOTA terminals [91]	AMBER 11	AMBER 11 ^ƚ^	GAFF and parm99	Location of DOTA groups complexed with Gd ions
PEgylated PAMAM G3 to G5 [92]	NA *	GROMACS ^§^	MARTINI	Effect of PEGylation on the structure and interparticle interaction
Pegylated triazine dendrimers linked with paclitaxel [72]	Material Studio 5	AMBER 11 ^ƚ^	parm99	Effect of PEGylation on availability of linkers
Carboxylic modified PAMAM G5 with gold, fluorescein isothiocyanate (FI) and folic acid (FA) [93]	Insight II	Insight II ^ƚ^	CVFF	Conformation and location of FI and FA
PAMAM G5 with amine, carboxyl and acetamide groups linked to fluorescein and folic acid [80]	Insight II	Insight II ^ƚ^	CVFF	Location of folic acid and its availability
PAMAM G5 with methotrexate [29]	CHARMM	CHARMM ^ƚ^	CHARMM	Location of MTX when directly linked or with a spacer
Poly(l-lysine) and Poly(amide) G4 dendrimers [94]	Starmaker (Silico)	NAMD ^§^	OPLS-AA	Comparison of dendrimer architectures in solution
Dendrimer-small molecule interactions				
PAMAM G5 with different terminal 81]	Insight II	Insight II ^ƚ^	CVFF	Influence of terminal groups on the complexation
PAMAM G4 + polyphenols [55]	ChemOffice Ultra 6.0	HyperChem Pro 7.0 ^ƚ^	MM+	Free binding energies
PAMAM G3 + nicotinic acid, nicotinate and 3-pyridiniumcarboxylate [95]	HyperChem	NAMD ^§^	CHARMM27	Free energy of binding and the effect of pH variation on binding
Peptide dendrimers + hydroxypyrene trisulfonate butyrate ester [38]	CORINA	GROMACS ^§^	GROMOS-96 43a1	Conformation and docking site location
PAMAM G5-Folic acid + Morphine and Tramadol [96]	ICM	NAMD ^§^	CHARMM 27; ParamChem	Different pHs; Binding mechanism elucidadtion; Location of folic acid
PAMAM G5 + salicylic acid, L-alanine, phenylbutazone, primidone [97]	DBT/AMBER	AMBER9 ^ƚ^	GAFF	Effect of pH on interaction and relation with drug release
Poly(l-lysine) G4 dendrimer + doxorubicin [83]	ChemBioOffice	Desmond ^§^	OPLS-AA	Complex formation
Dendrimer-nucleic acid interaction				
Triazine G2dendrimers + siRNA or DNA [98]	AMBER 10	AMBER 10 ^ƚ^	Parm99	Binding mechanism and energy contributions
PAMAM ssDNA [49]	AMBER 7	AMBER 7 ^ƚ^	AMBER 95 (DNA) DREIDING (dendrimer)	Binding interaction and energy contributions
PAMAM G3 DNA [75]	NA *	NAMD ^§^	CHARMM 27	Complexation mechanism
PAMAM G0 and G1 + siRNA [43]	Material Studio 5	AMBER9 ^ƚ^	Ff99 FF for RNA GAFF for dendrimers	Effects of pH on the complexation
PAMAM G7 + siRNA [99]	Material Studio 5	AMBER10 ^ƚ^	GAFF (non-standard residues); parm99	Complex interaction
Dendrimer-protein interaction				
Glycosylated PAMAM G3.5 + MD-2 protein [52]	XPLOR	Desmond ^§^	OPLS_2005	Docking and interaction between active and non-active forms
PAMAM G4 Albumin [47]	NA *	NA *	DREIDING	Contact points between dendrimer-albumin at physiological pH
PAMAM G0 with guanidinium terminal groups α-chymotrypsinogen A [73]	NA *	NAMD ^§^	CHARMM 27	Site of interaction with the protein and effect of salt types
Dendrimer-lipid bilayer interaction				
Acetylated and non-acetylated PAMAM G5 and G7 + DMPC [53]	Insight II	GROMACS ^§^	MARTINI and adapted MARTINI	Effect of size, charge and concentration on dendrimer-membrane interaction
PAMAM G3 and G5 with different acetylation levels + DPPC [60]	Insight II	GROMACS ^§^	MARTINI	Effect of size, charge and lipid phase on dendrimer-membrane interaction
PAMAM G3 with amine, acetyl and carboxyl terminals + DMPC [74]	CHARMM	CHARMM ^ƚ^	CHARMM27 (lipid) and CHARMM 22 (dendrimer)	Effect of terminal groups and lipid phase

The usefulness of DREIDING FF to predict the behavior of a PAMAM G4 dendrimer in solution was recently reported. This was the first report of a FF to be able to describe that the radius of gyration was independent from the pH and instead a reorganization of the internal structure occurs [85] (see Section 3 for more details). When compared to coarse-grain (CG) simulations, the Rg was found to be similar to those obtained by DREIDING FF. These studies confirmed the usefulness of the CG model when systems with larger length scales are simulated.

Finally, the surrounding environment should be defined as precisely as possible. This includes the presence of water molecules or other solvent, ions, lipid membranes or other molecules such as drugs. Although the dendrimer can be simulated in vacuum, this is not ideal due to the effects of solvent polarization on the structure. In the absence of explicit solvents and counter-ions, special care has to be taken since such approximations may lead to non-physiological conformations of the dendrimer. PAMAM G2 dendrimers were tested with different parameters to describe the implicit use of solvent and compare to the use of explicit solvent. It was observed that modifying the non-bonded cut-off distance and dielectric constant could led to radius of gyration different from those find with explicit solvent [87].

In simulation experiments, solvent molecules can be either modelled explicitly or implicitly. Explicit solvent calculations account for each individual water molecule, which is computationally demanding. Implicit simulations translate the behaviour of water into the forces experienced by the dendrimer. Although this introduces a simplification in the simulation, studies using both explicit and implicit solvents have shown good agreement [87,100].

Another way to simulate the solvent is through an intermediate approximation of a hybrid implicit/explicit solvation model. This approach uses explicit solvent only in the layer closer to the dendrimer ([35,101] for more information). This kind of approach was used to study the structural conformation of dendrimers in different solvents being able to reduce the computational costs and having an accurate solvation in the dendrimer interface [35]. After setting the parameters, the actual simulation is allowed to run. Several methods have been used but the majority of simulations performed on dendrimers have been (i) Brownian Dynamics (BD), (ii) Monte Carlo (MC) or (iii) molecular dynamics (MD).

BD uses simplifications that allow longer time-scale calculations. In these simulations individual dendrimers are treated as Brownian particles and evaluated for friction in the surrounding solvent (the flow) (reviewed [102]). This model was used to study the polyelectrolyte complexation between a charged G3 and G4 dendrimers and a linear polymer with the opposite charge. From these simulations it was observed that the polymer chain was adsorbed to the dendrimer in a higher amount than that required to neutralize the dendrimer [103].

MD and MC models are used to study the performance of dendrimers in biological systems including structural configuration and thermodynamic calculations. In general, dendrimers are defined with a starting configuration and then by assuming ergodic conditions, the system is minimized towards a low free energy. In this process, MC simulations use iterative random atomic displacements of the initial configuration to generate a new energy value which is then accepted or rejected by association of a probability function using Boltzmann statistics. This will depend on whether the study is performed in NVT (number of particles, volume and temperature constant) or NPT (number of particles, pressure and temperature constant) ensemble. The system is therefore evaluated to find a minimum configuration potential energy. By setting up constrains, the number of degrees of freedom can be reduced to decrease the computational demand. For this reason, allowing only important rotatable bonds to change is usually a good approximation.

Significant improvements for dendrimer analysis have been performed by MC parameterization using Continuous Configuration Boltzmann Biased direct Monte Carlo (CCBB MC) [35]. With MC, the dendritic chains torsional angles are sampled in a step-by-step process [35], conversely with CCBB MC they are sampled using a weighting function ([104]). These types of simulation, however, do not provide dynamic information. Dendrimers can be highly flexible and therefore the number of sampling conformations can be very high. In order to acquire information about dendrimer dynamics, which is very relevant in a biological environment, molecular dynamics should be used.

Molecular dynamics decrease computed simulation time (compared to QM) by introducing simplifications that assume that molecules interact as particles via classical mechanics of motion. In MD simulations, atoms interact over time by addition of impositions (temperature, pressure, time), restrictions (neglect of quantum mechanics and relativistic effects) and the integration of equations of classical mechanics (Newton mechanics, which represents the motion of the system). This allows following the trajectory of atoms with a high spatial resolution.

Before performing the actual measurement, energy minimization is advised. This can be achieved with a short simulation with restrictions to the degrees of freedom. For example, in the case of a peptide bond, these can be restrained to maintain planarity. At this point it is necessary to be careful so that the initial structure does not end in a local minimum, which can be difficult to reverse. An interesting approach to circumvent this was proposed by changing the partial charge to +0.1e of all atoms followed by an increase in temperature to 500 K. This promotes the extension of the structure to be evaluated [105]. Another approach that can guarantee that the dendrimer branches are the farthest away from each other can be the addition of NOE constraints during the assembly of monomers using XPLOR [71].

After minimisation, the equations of motion are used in iterative time-steps to simulate the dendrimer and the surrounding system of desirable conditions. Depending on the simplification of the model (e.g., all-atom simulation, coarse grained) different information can be obtained from the simulations. All-atom simulations can be performed with dendrimers but might require high computational resources. This type of simulation gives relevant information including the 3D configuration and atomistic detailed interaction events.

Coarse-grained simulations can offer greater simplification allowing the system size and the simulation time-scale to be increased while still providing significant realistic details [42]. This can be accomplished by a reduction in the number of degrees of freedom. By defining an “atom” that represents an average of *n* atoms (generally four non-hydrogen atoms) the simulation demand is decreased. This allows a broader range of simulations to be performed, but at a cost of losing an accurate reproduction of structural details (lower resolution). For example coarse-grained simulations do not provide information about hydrogen bonding [42]. Nonetheless, this method is still used as a valuable tool since it has shown to be valid for large systems including lipids and dendrimers (Section 3.3). As a result of reducing the number of particles and the number of degrees of freedom, the time-step can be greatly increased.

After performing a simulation, there are particular features that can be obtained and analysed. These include the radial distribution function (RDFs), solvent accessible surface area (SASA) and solvent excluded volume (SEV), radius of gyration (Rg), shape descriptors, counting number of hydrogen pairs, and the mechanistic interactions as well as the thermodynamic parameters associated with them. Together these features can provide a profile of a given dendrimer.

RDFs are especially interesting to evaluate dendrimers as drug delivery systems since they provide additional insight into the distribution of all the constituents of the system. A peak in this kind of representation indicates the distance from the centre of mass (for example) at which atoms remain in a locked conformation for a long time. In contrast a diffuse distribution can either mean a homogeneous distribution throughout the area of interest or a molecular movement. RDF can therefore be used to study the distribution of atoms such as water molecules, ions and drugs within the dendrimers’ interior. For example, the distribution of Mefenamic acid in PAMAM G2 and G3 revealed that both dendrimers were able to encapsulate it through internal and external interactions giving an estimate of the number of molecules involved in these interactions [106]. It also measures the terminal group distribution which can be highly valuable to study the exposure of specific groups with targeting functions [72]. In this regard, the effect of surface groups was studied on the radial distance of folic acid from the center of mass. This study showed that depending on the dendrimers’ surface group, the folic acid would be more or less exposed to the surface [80]. This can also be used to define the hydrodynamic radii of the solvated dendrimer by analyzing the solvent around [38].

SASA and SEV also give valuable information about dendrimer structure. These parameters allow the determination of geometrical shape, available non-solvent accessible internal space and the accessibility of groups of interest to the solvent (solvent accessible volume). As an example, if the release of a moiety is sensitive to the solvent, this information can be used to design dendrimers that can effectively burry or expose this molecule. Such case was studied by MD for the availability of labile linkers on different PEGylated dendrimers, to see if the linker was available, and could therefore act as a prodrug [72]. SASA and SEV examine the molecular surface with a spherical solvent probe to roll around the van der Waals spheres of the macromolecule. This modeling strategy can also provide information related to the internal cavities [107] and can be used to estimate how many molecules the dendrimer will be able to carry. This is particularly useful to characterize different types of dendrimers and compare which one has the highest potential. This strategy was applied to two families of denamide and denurea dendrimers where the measurement of the internal cavity size and volume was used to estimate the theoretical number of molecules that would fit into these cavities [107].

Finally, the Rg, which is correlated to the size, allows the comparison of simulation data with the experimental data and therefore the validation of the simulation model. When simulation values are similar to the experimental ones, it is expected that the simulation forces are well described. The analysis of Rg values can provide insight into the swelling or shrinkage of the dendrimer in the different conditions [85]. In particular several simulations have been performed on PAMAM dendrimers at different pH and salt concentrations to understand its behaviour in solution (see Section 3.1). The radius of gyration can also be calculated from the gyration tensor to provide insight into the shape of the dendrimer. In this case, several ratios between the various components of the gyration radius (in the x y z direction) can inform on whether the dendrimers is shaped like a sphere or more as an ellipsoid [105].

### 2.3. Molecular Docking of Dendrimers

Before performing the actual docking of drugs into the structure, it is possible to estimate the potential of the dendrimer architecture to accommodate the drug inside. To illustrate this, two types of dendrimers (oxy-urea and oxy-amide groups) with varying branch lengths were evaluated for their cavity sizes that were available for host interaction with anti-parasitic drugs [107]. Equilibrated dendrimers were obtained from MD and the cavity dimensions in the interior were estimated from the difference between the van der Waals volumes and the solvent-excluded volumes. Since the size of the probe can over-estimate the molecular dimensions, an adequate probe size was first determined on the slight inflexion of the SASA curve* vs.* sphere radius. Measurements of cavity size were shown to be dependent on the increase of the generation but not significantly for branches derived from aliphatic chains. Also, oxy-urea dendrimers were less porous than oxy-amide, seemingly due to intramolecular interactions [107]. The oxy-amide dendrimer was found to be more adequate for the incorporation of molecules since it was more flexible and had more adequate size of cavities.

The measure of the cavity size can also be used to quickly estimate the maximum number of drug molecules that can be incorporated into the dendrimer [54]. However, these do not account for the favorable or unfavorable interaction energies that may occur between the dendrimer and candidate drugs. For this reason docking scores can be used to calculate free energies of individual binding sites. Modified PAMAM dendrimers were blindly docked with curcumin in Autodock using a grid box with 0.3750 Å spacing. Experimentally it was found that this dendrimer could accept up to 5 molecules and thus the most favorable energies calculated by the docking score (in the range of 4.04 and −7.28 kJ·mol^−1^) were attributed to be the binding locations [108].

The alternative approach can also be used in which the docking conformations are first prepared to give the starting point structure for MD simulations. In this case the drugs can be placed in the interior of the dendrimer instead of randomly placed near the dendrimer with the hope that the drug will find the docking site along a trajectory in a short period of time. This is difficult to accomplish for large systems given the density of monomers on the interior. However this approach can prevent the possible clashing of atoms, which can occur when attempting to manually introduce the drug into voids of the dendrimer.

Using this random approach, four different drugs (salicylic acid, L-alanine, phenylbutazone and primidone) were docked to the interior of a PAMAM G5 where the grid box was limited to the center region in AutoDock Vina [97]. The best docking score conformations for each drug were then used as initial structure for further MD simulations with AMBER with explicit water as solvent. Another interesting way that has been reported to incorporate drugs into dendrimers is to artificially create cavities inside the dendrimer. This can be accomplished by applying a force to a select number of atoms and then inserting the drugs into those cavities with common docking software such as AutoDock [109].

**Figure 6:**
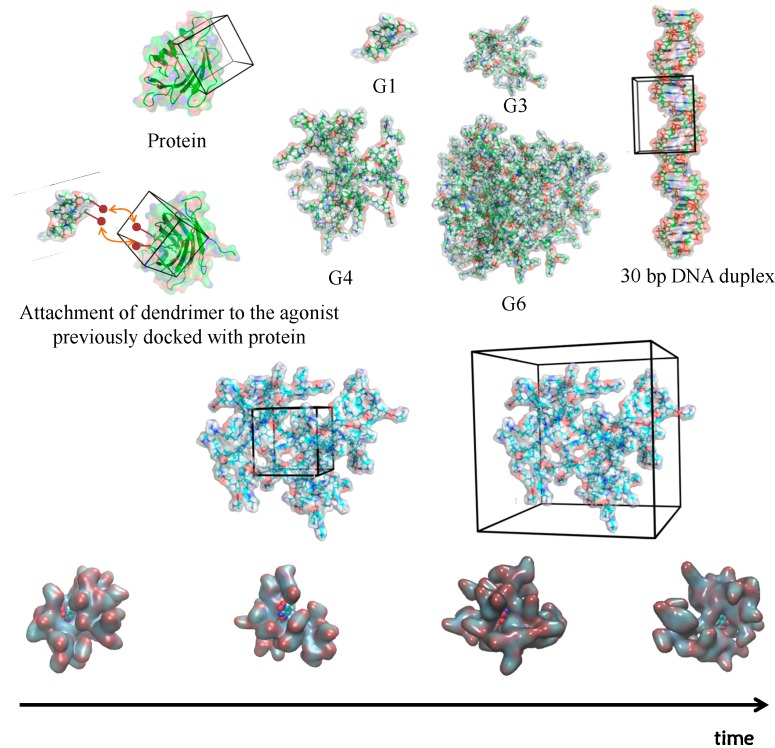
Docking strategies applied to dendrimer studies. Depending on the size either the dendrimer or the biomolecule can be described as the docking center. The docking site can be defined either in a small space of the dendrimer or in the whole structure (middle figures). The generated docking structures can then be submitted to MD simulations or the conformations from MD simulations can be used to dock the molecules (Bottom figures).

Dendrimers can also be treated as the ligand instead and the receptor can be a protein. The protein model of A2A receptor homodimer linked to an agonist (CGS21680 molecule) was used as reference to study if multiple copies of this agonist linked to a PAMAM G3 would be able to simultaneously occupy both subunits of the receptor. To create this kind of docked molecule, a small part of the dendrimer was first docked into the protein and then the rest of the dendrimer was attached with the favorable docked agonists already in place (see Figure 6). This created some clashes of overlapping branches with the protein which were then manually adjusted followed by minimization and subsequent MD simulation [48].

In contrast to this approach, the full size partially glycosylated and non-glycosylated (negative control) PAMAM dendrimers G3.5 were used as ligands in rigid docking against human MD-2 glycoprotein as a target. This was achieved using both Patchdock and Hex software for docking. Since both software packages were based on rigid docking, twenty different dendrimer conformations were obtained from MD. The partial glycosylation was found to promote better shape complementarity and showed a higher number of interactions compared to the non-glycosylated form. The docking interaction calculated with HEX showed that the partially glycosylated dendrimer co-operatively interacted with residues in the MD-2 entrance pocket revealing that not only shape but more importantly the electrostatic interactions were crucial for the biological activity of these dendrimers. The docked structures were then used as base for the MD simulations [52].

These examples illustrate the usefulness of docking methods to estimate free energy bindings, identify binding site locations and even to explore the potential of a dendrimer as a therapeutic agent. In fact, docking can be used as a preliminary filtering tool to optimize dendrimer structure. Specifically, it can be used to determine the potential use of a dendrimer to bind a guest molecule. Furthermore, if the* in vivo* behavior of the dendrimer is known and is interesting, docking methods can be used to evaluate a potential range of drugs that would fit the desired* in vivo* profile. Docking can be used to check if drugs can be incorporated into the dendrimer in a similar process that is used in high throughput *in silico* screening performed for protein targets.

## 3. Modeling Dendrimers for Biomedical Applications

The design and development of dendrimers for biomedical applications is accelerated when there is an understanding of some of their properties in a physiological milieu. Molecular modeling techniques can provide the means to gain an increased understanding of important molecular structural features and dynamic behaviours that are fundamental to a biomedical application. Many methodologies have been developed and validated to model these molecules with good correlation with experimental results.

### 3.1. Impact of Solvent and Dendrimer Topology

One of the primary applications of molecular modeling for the study of dendrimers has been to better understand conformational behaviour in solution. As expected dendrimer structure and topology appears to be dependent on the factors inherent to the dendrimer (generations, monomers length and their chemical properties) and factors related to the solvent (type of solvent, salt type and ionic strength) [107,110]. The combination of these factors affects the binding pockets to establish host-guest interactions as well as the possible moieties at the surface of dendrimers.

There has been a particular emphasis to understand the behaviour of PAMAM dendrimers in solution. This class of dendrimer is commercially available and there is vast array of experimental data that can be used for comparison. As a result, different levels of theory and force fields have been applied to PAMAM dendrimers from which Rg values have been measured and compared to experimental data (see Table 2). Although PAMAM dendrimers can have limited biomedical applications due to their inherent toxicity at higher generations (see [31]), these studies are important for methodology development and validation. Notably, PAMAM dendrimers exhibit different protonation levels at different pH value since they are composed of both primary and tertiary amines. Experimentally, it has been determined that for G4 [111] and G8 [112] the size of the dendrimers is independent of the pH with a variation of 4% and 2% respectively.

Since representing water and ions explicitly can be computationally demanding, the use of lower levels of theory is often considered and the solvent is treated implicitly. A way to overcome this problem is to perform an all-atom simulation with explicit solvent on a smaller generation and compare the radius of gyration and atom distribution with simulations performed with different implicit parameters [87]. From such studies it was found that the use of a distance-dependent dielectric constant without a cut-off distance had the best similarities to the explicit simulations for the neutral and low pH dendrimers and was reasonable for the high pH. However, when comparing the values of Rg obtained for a G4 dendrimer, this model was not able to predict the size invariability, previously was determined experimentally at different pH values [111]. One key aspect is that the implicit treatment of water may not fully represent the solvation to the necessary degree, including the diffusion of water molecules inside the dendrimer branches that contribute to swelling [110].

A good solvent system is essential for a reliable prediction of dendrimer size and conformation, and the evaluation of solvent penetration into dendrimer void spaces [113]. The water behaves differently depending on its position to a dendrimer. In the case of PAMAM dendrimers three classes of water have been described: (i) buried water; (ii) surface water at the dendrimer-solvent interface and (iii) bulk water (*i.e.*, solvent) [113,114]. Water molecules are enthalpy favored near the dendrimer and buried water has lower entropy in relation to the bulk water. Therefore, the binding of a water molecule to a dendrimers molecule results in release of this difference of free energy [114].

Water molecules penetrate inside the dendrimer and compete for H-bonds between dendrimer residues [89,110]. However, other factors such as the force field employed may contribute to the correct prediction of experimental data (see Table 2). Recently, a study using all-atom simulations of PAMAM G4 with explicit solvent and counter-ions employing the DREIDING force field with optimized parameters (e.g., hydrogen bonding Cl^−^-^+^HN(CH_3_)_3_) [85] was able to predict the behaviour of these dendrimers at different pH. These simulations were not only able to predict the low variability of Rg (swelling of 4.9%) at different pH values, but they also elucidated the conformational mechanisms that the dendrimer underwent. It was observed by measuring the radial density distribution that the dendrimer underwent an intermolecular transition from a “dense core” at high pH to a “dense shell” at low pH. This mechanism of mass redistribution is important to interpret for example the encapsulation and the possible release mechanism of drugs from such dendrimers. This kind of behaviour of conformational change triggered by pH changes is highly desirable for intracellular delivery and is critical for the development of stimuli responsive polymers.

Different types of solvent can also promote structural modifications of the dendrimers that impact their properties. MD simulations were conducted in explicit solvent for dendrimers with a linear PEG chain in the core. Depending on the solvent (methanol* vs.* THF) the PEG was more extended or more compact in order to increase or decrease the interaction with the solvent. This resulted in burying of the dendrimer [35]. Although the solvent accessible surface area (SASA) was not measured for these dendrimers, the measurement of the radius of gyration revealed that in methanol the PEG core extends outwards and tends to wrap around the dendrimer as observed in the snapshots from the last frames of simulation. This study provided rationale for experimental results [115] where two structure forms were suggested (wrapping around* vs.* loops to the exterior). This is important as it gives a mechanistic view of the changes of the material in different solvents and can be used design dendrimers that respond differently in different solvent systems. Likewise, a PAMAM G5 dendrimer with amine terminal groups and 90% acetylation was simulated in explicit water and methanol using the General Amber Force Field (GAFF) in the presence of counter-ions. Measurements of the radius of gyration showed that the presence of acetylation promoted the dendrimer to shrink and become more compact compared to the non-acetylated dendrimer. The size of both dendrimers were similar to what was observed experimentally (2.50 nm (SEC)* vs.* 2.51 nm (MD) for G5 PAMAM and 2.35 nm (SEC) *vs*. 2.11 nm (MD) for PAMAM G5 acetylated and in methanol 2.41 (SAXS) *vs*. 2.57 nm (MD) for PAMAM dendrimer [89]).

**Table 2:** Non exhaustive list of PAMAM dendrimer simulations and their radius of gyration. For comparison the experimental values obtained by SAXS and SANS are displayed on top.

FF	Solvent	G0	G1	G2	G3	G4	G5	G6	G7	G8	G9	G10	Source
**SAXS**	Methanol				15.8	17.1	24.1	26.3	31.9	40.3	49.2	57.4	[86]
**SAXS**	Methanol	4.0	7.9	11.8	15.09	18.60	23.07	27.50	32.11	38.58	-	-	[116]
**SANS**	D2O	-	-	-	-	20.90–21.30	-	-	-	-	-	-	[117]
**SANS**	D2O pH 4.97–10.25	-	-	-	-	20.64–21.58	-	-	-	-	-	-	[111]
**SANS**	D2O pH 4.7–10.1	-	-	-	-	-	-	-	-	38.1–40.7	-	-	[112]
**SANS**	Different solvents	-	-	-	-	-	22.1	-	-	32.8–43.8	-	-	[118]
**DREIDING**	Vacuum	4.93	7.46	9.17	11.23	14.50	18.34	22.40	29.09	36.42	46.03	55.19	[10]
**DREIDING optimized QM**	Water explicit	-	-	-	-	21.07–22.11	-	-	-	-	-	-	[85]
**DREIDING**	Water implicit	4.97	7.03	9.77	13.01	16.36	21.67	27.62	-	-	-	-	[35]
**DREIDING**	Water explicit High pH	-	7.4	11.5	12.9	16.9	20.3	24.7	30.1	-	-	-	[119]
**DREIDING**	Water explicit Low pH	-	9.4	13.6	17.2	21.1	26.1	32.5	37.57	-	-	-	[119]
**CHARMM 27**	Water explicit	-	-	-	15.33	21.04	25.50	30.18	-	-	-	-	[95]
**AMBER**	Water explicit pH 7.4	-	-	-	16.25	18.8–20	22.43–22.9	27.2	-	-	-	-	[50]
**AMBER**	Water explicit pH 5	-	-	-	-	21.0	24.2	28.9	-	-	-	-	[50]
**DREIDING**	Water explicit pH 4–12	-	-	-	-	-		-	-	37.8–43.11	-	-	[120]
**Coarse-Grained (MARTINI)**	Water explicit	-	-	-	-	20.1	25.6	-	-	-	-	-	[59]
**Coarse-Grained**	Water explicit	-	-	-	13.1	-	23.20	-	-	-	-	-	[60]
**DREIDING**	Water explicit pH 12	-	-	-	-	16.78	20.67	26.76	-	-	-	-	[110]
**DREIDING**	Water explicit pH 7	-	-	-	-	17.01	22.19	27.28	-	-	-	-	[110]
**DREIDING**	Water explicit pH 4	-	-	-	-	19.01	24.76	30.89	-	-	-	-	[110]
**CVFF**	Water implicit Low pH	-	-	16.6	22.8	29.9	38.0	46.8	-	-	-	-	[87]
**CVFF**	Water implicit Neutral pH	-	-	14.5	19.7	26.7	32.8	41.3	-	-	-	-	[87]
**CVFF**	Water implicit High pH	-	-	8.4	11.6	14.8	18.3	24.2	-	-	-	-	[87]
**OPLS**	Vacuum	-	-	11.0	13.7	-	-	-	-	-	-	-	[121]

Although water molecules led to more hydrogen bonding between the dendrimer and the solvent, the number of intra-molecular hydrogen bonds was found to be similar both in water and methanol. From the snapshots used to illustrate the H-bonds in the dendrimer, several cis-conformations of the amide bond can be observed. This could have resulted from not setting constraints to *trans* configuration while generating the dendrimer. Because of the large size of the dendrimer it would be hard to return to the most stable conformation. The results of this effect on the overall structure cannot therefore be assigned. Measurements of the relative shape anisotropy showed that both dendrimers correlated well the spherical model and that it was not affected by the solvent [89].

A combination of the effect of the solvent and the topological features of dendrimers was studied using coarse-grained MD with different dendrimers’ generations (G4 to G7), different spacer lengths (1 to 6 molecules) and charges (neutral, partially and fully charged). From these structures it was observed that neutral dendrimers had a more spherical and compact structure compared to charged dendrimers, which had void spaces in their interior. It is expected that the resulting space left would be available to encapsulate drugs. It is also expected that with increasing size, higher volume will be obtained. Experimentally it was found that the increase in PAMAM generation from 4 to 6 increased the internal volume [122], which could also be observed by the measurement of solvent excluded surface of the simulated dendrimers [110]. Modifying the size of the spacer in the core was also observed to have an enormous effect on the size of the dendrimer as well as on its internal structure which changes from a compact almost sphere to a “blob-like” structure [123].

Another example where modeling can be crucial for designing dendrimers as potential drug carriers was studied in two families of an asymmetric (poly(L-lysine) and symmetric cationic G4 dendrimers at different pH. Both dendrimers were built in Starmaker and parameterized using the OPLS-AA FF. The simulations were performed with explicit solvent and counter-ions in NAMD for 10 ns. Both protonated dendrimers exhibited extended conformations (40% and 60% higher Rg for the SPAM and PLL respectively) whereas, in the neutral state a significant back-folding of terminal chains was observed [94]. The PLL dendrimer showed a higher deviation from the extended spherical form showing that the asymmetric nature of the dendrimer might facilitate other type of conformations.

Predicting this kind of “smart” or stimulus triggered behaviour has tremendous impact for applications as drug delivery systems since new dendrimers can be designed to encapsulate drugs in specified conditions (e.g., pH or other type of solvent), which then* in vivo* prevent the burst release from the entrapped drugs inside. It can also potentiate the design of a stimuli-responsive mechanism such as pH-triggered release, which can be useful for intracellular release.

### 3.2. Impact and Versatility of the End Groups

Because of the hyperbranched topology of dendrimers, there are many end groups that can be tailored to participate in multiple specific or non-specific interactions simultaneously. A common practice for overcoming the potential toxicity of multiple charges at the surface has been the attachment of other groups such as acetyl, PEG or lipid moieties. The end groups can also be modified to carry targeting moieties, e.g., folic acid [80].

**Figure 7:**
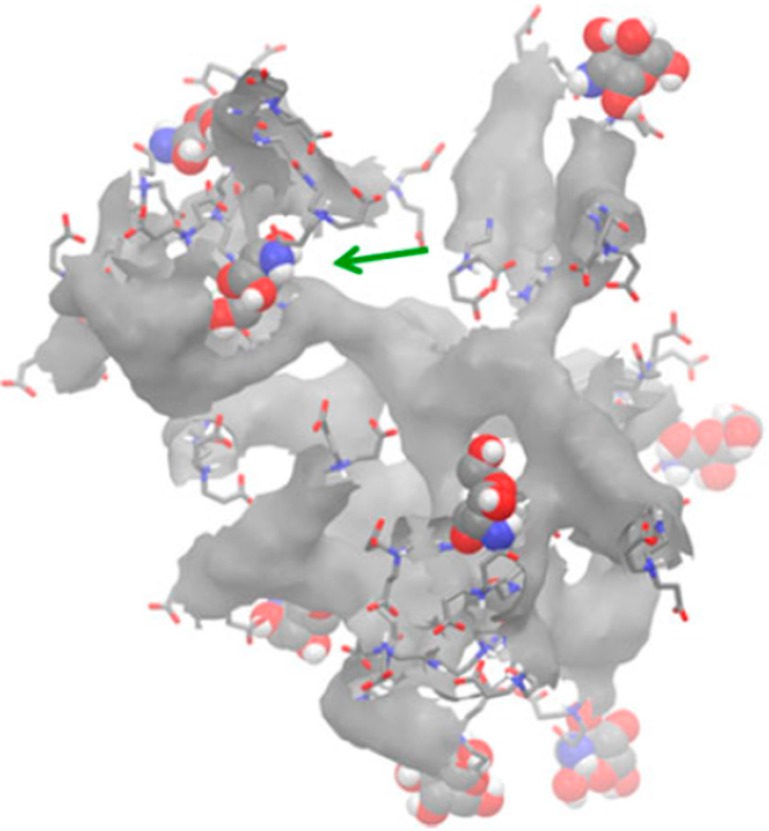
Availability of the substituted terminal glucosamine groups on PAMAMs’ surface. Reprinted from [90].

End group modifications can influence the overall dendrimer structure resulting in altered host-guest interactions or inefficient display of the targeting moieties. As an example, a common observation for PAMAM dendrimers is the back-folding of the terminal end groups [110,120,124] as measured from the density distribution of the terminal groups from the centre of mass. If the intended goal is to protect a labile molecule from the external environment, this type of structure would be effective. However, in the case of a linked moiety (e.g., targeting molecule) this kind of structural arrangement would, most likely, be ineffective (see Figure 7). Both cases can be elucidated by molecular modeling, which help with structure design. Modeling approaches can sample the location of these groups and their available surface area to the solvent and provide some insight about the potential of modified end groups to be available to interact with a biological target.

With the intent of examining the effect of terminal capping groups on the exposition of the folic acid to interact with the receptor, MD simulations were carried with CVFF in implicit solvent treated with a distance-dependent dielectric constant [80]. PAMAM G5 dendrimers with folic acid and terminal groups composed of amine, hydroxyl, carboxyl and acetamide, simulations showed that both dendrimers with charged groups internalized folic acid groups as measured by the mean distance of folic acid from the centre of mass and compared to the Rg. In contrast, in the case of the acetamide derivative the surface groups were extended away (mean distance of 26.7 Å* vs.* 19.8 of the Rg) suggesting potential capacity to interact with the receptor.It was suggested that the hydroxyl terminated dendrimer had higher exposition of the folic acid to the surface (mean distance of 27.2 Å* vs.* 21.8 of the Rg), although not as pronounced as in the case of acetamide derivative. Altogether these measurements correlated directly with the cell internalization assay of these dendrimers to KB cells [80].

Similarly, all-atom simulations with CHARMM 22 force field on PAMAM G3 terminated either with amine, hydroxyl groups or hydroxyl groups with four methotrexate (MTX) molecules were tested for the availability of MTX. The measurement of Rg for PAMAM-OH and PAMAM-MTX was smaller compared to PAMAM-NH2 (13.1, 14.0 and 18.8 Å respectively). Although more collapse topologies (in comparison to PAMAM-NH2) were obtained, the snapshots of the equilibrated simulations indicated that the MTX remained at the surface. This result correlated positively with the binding affinity of these dendrimer found experimentally [125].

Considering a different approach, MD simulations were conducted to design dual labelled dendrimers in which the probes do not interfere with one another (by quenching or any other way) [126]. A PAMAM dendrimer was functionalized with both carboxy-fluorescein and tetramethyl-rhodamine. PEG spacers were introduced in order to study if it was able to maintain both fluorophores at a certain distance. The simulations were carried out in AMBER 11 with either no PEG or 44 monomers of PEG and an explicit water model was used with enough ions to neutralize the charges of the dendrimer together with 150 mM of NaCl to mimic experimental conditions. The analysis of the radial distribution function and fluorescein-to-rhodamine distance both suggested a suitable distance between dyes only in the presence of the PEG spacers. These results were in accordance with the quantum yield measured by optical spectroscopy [126]. Based on the validation by the modeling studies these dendrimers were used to probe the physiological/pathological microenvironments by measuring the fluorescence and making the assumption that both probes did not interfere with one another.

This type of approach has good potential to develop diagnostic tools. A practical and very beneficial application of this kind of molecular modeling design of dendrimers with several types of sensing probes would be for example in guided surgery by luminescence of tumour cells [127]. Dendrimers could be design to have sensing probes with targeting groups to selectively be internalized by cells. The probed would light up differently depending on whether or not they are tumour cells. This difference would help to identify areas surrounding the removed tumour and therefore a more efficient removal would be obtained instead of removing healthy tissue surrounding just for precaution. This approach would also be useful to identify metastasis that would not be identified by other methods [127].

Poly(ethylene glycol) (PEG) chains are also commonly used in biomedical applications to stabilize macromolecules (e.g., proteins) and increase their half-life. Coarse grained simulations with MARTINI FF in GROMACS were used to study PAMAM dendrimers of different generations linked to PEG chains of different size in order to study the conformation and aggregation of these dendrimers in solution [92]. Although CG simulations use a lower level of theory the measured Rg values was in close agreement to those measured experimentally (theoretical values: 5.98, 6.65 and 740* vs.* 5.67, 7.06 and 7.67 nm measured experimentally) reassuring the usefulness of the predicted model. Altering the size of PEG chains in the PAMAM surface showed remarkable differences. When higher MW PEG chains were used, a completely coverage of the dendrimer was achieved and, although PEG tends to extend towards water, the spherical characteristics of dendrimers was maintained. Simulations up to 400 ns of binary systems of both lower and higher MW PEG chains revealed that no aggregation between two dendrimers was observed as measured by the distance of both centres of mass [92].

This interesting approach allows the study of the impact of surface modifications (e.g., linkage of targeting groups such as antibodies to the end of PEG chain) and the prediction of dendrimer behaviour to aggregate in solution that would make them ineffective. This kind of approach was also useful for the design of dendrimers with bio-labile linkages that were available to the solvent so that the degradation could occur [72]. Triazide dendrimers with paclitaxel were linked to different number of hexaPEG, nonaPEG or dodecaPEG and simulated in AMBER with parm99 all-atom force field. These simulations were performed to ensure that the bio-labile linker was available at the surface so that it could be degraded and release the anti-cancer drug. Measuring the RDF it was found that increasing the PEGylation lead to a significant increase of PEG at the location of the linker [72]. Nevertheless, for all types of PEGylations, paclitaxel was found to be homogeneously distributed and close to the surface with only a small number of molecules back-folding.

Using a different approach, G5 dendrimers with different terminal groups, at different pH values, were build using Insight II software and simulated using CVFF to correlate the conformation of the dendrimer with the release and efficacy of an anticancer drug [81]. For this, seven 2-methoxyestradiol (2-ME) molecules were randomly incorporated in the simulation box. The position of 2-ME was measured from the centre of mass and was found to be farther for G5 with amine terminal groups and N-Glycine-OH. Except for G5-carboxyl all other structures exhibited open structures, which could be attributed to the release of the drug. These findings were consistent with the lower toxicity of G5-carboxyl observed in KB cells as the collapse structure would not allow the release. Although only a short simulation of 100 ps was performed the consistency with the structure obtained was achieved. However, the authors did not provide rationale for the superior encapsulation of 2-ME by ACE terminated dendrimers found experimentally, which might be caused by the aggregation of 2-ME inside the dendrimer that is neutral at this pH with the relative collapse of the structure in comparison to amine and carboxylic terminated dendrimers [81].

### 3.3. Dendrimers Interaction with Lipid Membranes

Despite the tremendous developments of methods to study dendrimers, it is still a great challenge to develop novel dendrimers. Regardless of the applications for which dendrimers are used in nanomedicine, the fundamental knowledge of how these interact at the interface with cells is mandatory. Designing smart entities able to deliver their therapeutic payload to the site of action and circumvent physiological barriers is a promising strategy to achieve better therapeutic efficacy. Dendrimers interact with cells, and more specifically with the lipid bilayer. Cellular membranes are complex systems and a number of techniques have been used to address the interaction of dendrimers with these biomolecules including AFM, DLS, NMR, DSC and Raman Spectroscopy [128,129,130,131,132]. However, this is a difficult task, in part due to the extreme complexity of biological membranes. They contain a tremendous number of different lipids and proteins able to modulate the interaction between the dendrimer and the membranes.

**Figure 8:**
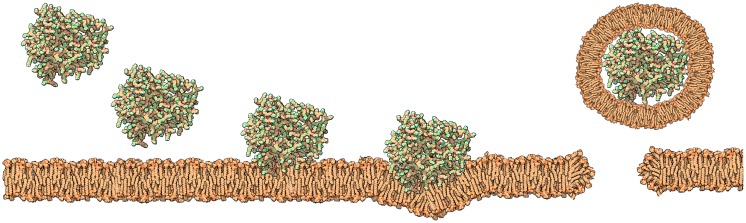
Proposed mechanism for high generation of PAMAM dendrimers; there is an initial attraction to the membrane governed by several forces (e.g., electrostatic); once the dendrimer is near the membrane the inner leaflet promotes interactions with the dendrimer. For high generation, the dendrimer is not able to flat and causes perturbation on the membrane leading to the formation of a vesicle encasing the dendrimers with subsequent formation of a pore.

Nevertheless, the results observed for model membranes have been well correlated with toxicity observed on cells. It is generally acknowledged that polymers with high density of cationic charge are more likely to cause disruptive effects on membranes [133]. In the case of PAMAM dendrimers these were found to have high deleterious effects both in model membranes [128,129] and cells [134,135]. In fact they were found to cause toxicity in a concentration, terminal charge density and generation dependent manner [136]. During this interaction, either the dendrimer creates holes (Figure 8), disrupts the membrane (particularly at higher generations) or is well accommodated in the lipid bilayer. Although this effect may mediate a higher cell transfection or even be useful as an anti-microbial activity [82,137] it is not suitable for drug delivery systems. Despite the experimental data that has been obtained, molecular dynamics simulations have been useful to elucidate the molecular mechanisms underlying these results [37,74,138]. In fact, molecular simulations allow testing hypothesis that would be impossible to test experimentally. As an example, simulations of cationic G5 and G7 dendrimers of different concentrations on a DMPC membrane were tested with either short or long PME electrostatic interactions [53]. For the short PME electrostatic range no bending or insertion of the dendrimers was observed, suggesting that the long-range electrostatic interactions (*i.e.*, interactions with the inner layer of the membrane) are indeed necessary for prediction of dendrimer toxicity.

Until recently, molecular dynamics using all atoms and explicit solvent systems have been restricted by the size of the system. Although there has been great progress in computational sciences, this type of simulation is still limited in terms of timescale. Coarse-grained models loose some finer details such as hydrogen bonding but they provide an alternative to full atomistic simulations. Recent reports show they can successfully be used to generate predictive models of dendrimer-membrane interaction [53,77,139].

In general terms, from the various simulation studies, dendrimers first interact with lipid membranes through different forces and depending on their composition, size and concentration, as well as, the properties of the membrane, different phenomena can occur [60,74,77,138,140].

In order to settle the influence of size on the disruptive mechanism of PAMAM dendrimers in the lipid bilayers, coarse-grained simulations of G3, G5, G7 and G9 were performed with different tensionless membrane models (DMPC and two membranes with shorter and larger tails). The radius of gyration measurement showed that G3 and G5 dendrimers were able to flatten upon interaction of interaction with the DMPC membrane [77]. This effect has been widely observed in simulations including in all-atom simulations for differently terminated G3 dendrimers (amine, carboxyl and acetyl groups) [138] and has been attributed to the attempt of the dendrimer to maximize the interactions of all branches. However, at higher generations the flattening process upon interaction with the membrane is less evident due to the density of branches making it thermodynamically unfavorable [141]. As a result, the dendrimers keep their globular rigid conformation resulting in the pulling of the membrane instead. Upon interaction with the membrane, no pore formation was observed with for the PAMAM G5 in DMPC membrane [77]. This is clear agreement with experimental data where PAMAM G5 was only able to expand existing defects but does not form new ones [133]. The measurements between the dendrimer and the membrane in the z-axis were evaluated as a measurement of dendrimer permeability. Dendrimers G3 and G5 showed small values consistent with the adsorption model in the membrane for all types of membranes. On the opposite side, G7 and G9 values were consistent with the embedded model in the membrane. The permeability also increased with the decrease in the tail size. From the snapshots, presented by the authors in the original publication, it is possible to observe significant distortions induced in the DMPC membrane while for shorter tail size membranes the G7 and G9 induced the formation of dendrimer-filled vesicles. On the contrary, for larger tail sizes the distortions induced by these dendrimers were smaller [77].

Dendrimer concentration has been other factor that showed to increase the toxicity of PAMAMs, experimentally. To explore this effect on membranes, a PAMAM G7 and G9 on a DMPC membrane were simulated using different simulation box sizes. These results showed that by increasing the area of the membrane, the cavities induced by the dendrimer became smaller [77]. However, this type of “dilution” may not capture the collective behavior of more than one dendrimer in the near space (higher concentration) as it only considers one dendrimer at the time. To overcome this, simulations of both positively charged and acetylated dendrimers were simulated in clusters in a DMPC bilayer [53]. An interesting observation from these simulations was that only 4 positive PAMAM G7 dendrimers were necessary to induce a strong bending on the membrane with insertion of some branches and pore formation while it required 16 positive G5 to induce some bending and insertion to the membrane. Furthermore, these effects were not observed for acetylated G5 dendrimers but these dendrimers aggregated instead [53]. Since the terminal groups can have such impact on dendrimers interaction, all atom simulations with implicit solvent were performed. A smaller system of a PAMAM G3 dendrimer with different terminal groups (amine, acetyl and carboxylic groups) on a DMPC bilayer were used to study the energy component involved in these interactions. The free energy binding was found to be 47, 36 and 26 kcal/mol for the PAMAM-carboxylic, PAMAM-amine and PAMAM-acetyl respectively and the attractive force was similar for both charged dendrimers [137]. These results are expected due to the zwitterionic character of DMPC lipid and therefore charged dendrimers interact more favorable than acetylated neutral ones.

Recently, a full all atom simulation was performed with a PAMAM G3 with amine terminal groups on a DPPC membrane and a discrepancy of 15 kcal/mol larger over the previous simulation was found. This result suggested that the explicit treatment of water significantly affects the adsorption thermodynamics as it contributed to the more entropic favorable interactions by releasing water molecules while pushing the dendrimer out of the water phase [142].

Lipid aggregation can also lead to the formation of fluid and gel phases. Experimentally AFM measurements of PAMAM G7 showed that the disrupting mechanism was abolished in gel phase of DMPC membrane [143]. Coarse-grained simulations of PAMAM G3 and G5 were simulated in DPPC bilayer at different temperature (277 K and 310 K) to simulate a more ordered phase compared to a more disordered phase. The results showed that during the simulation time no insertion of PAMAM G5 was observed at 277 K, contrary to the insertion observed at 310 K [60]. Similarly, all atom simulations of PAMAM dendrimers differently terminated (amine, carboxyl and acyl groups), with implicit solvent treatment, tested the fluidity of the membrane on dendrimers interaction. In this case, the gel phase was simulated by immobilization of the lipid tails from an equilibrated fluid phase DMPC bilayer. In the case of the fluid membrane a depression was formed to accommodate the dendrimer while the dendrimer flatted to extend the number of interaction. On the contrary in the gel phase the dendrimers remained at the surface without inducing any kind of deformity.

Cell membranes, however, are much more complex than a simple unique lipid bilayer. For instance they are composed of different lipids and the combination of different lipids can lead to different properties. To address this issue, a recent study using coarse-grained description of three types of lipids (DPPC, DPPE and DPPS) were tested in different ratios (to mimic erythrocyte membrane) with G4 and G5 PAMAMs [59]. The asymmetry of the membrane showed to be correlated with the ability of the dendrimer to insert on it. In the case of symmetric membrane, PAMAM G4 remained at the surface of the outer leaflet. These differences were attributed to the electrostatic attraction between the inner leaflets of the membrane towards the dendrimer. Increasing the percentage of DPPS from 10% to 50% showed a decrease in the distance between centre of mass between dendrimer and the membrane, meaning that the insertion of the dendrimer was higher. Complementarily, the order parameter of the phospholipid tail (a measurement of the movements of lipid bilayers) revealed that the incorporation of the dendrimer decreased the lipid order. The PAMAM G5 created more perturbation in the lipid order that resulted in an observed the transient formation of a pore [59]. The type of lipids on the membrane can also have a specific role on the interaction with dendrimers. Using coarse-grained simulations of PAMAM G3, G4 dendrimer in bilayers composed of DPPC mixed with dipamitoylphosphatidyl-glycerol (DPPG) it was found that the dendrimers promoted the diffusion of the oppositely charged DPPG lipid from the inner to the outer leaflet [144,145]. This formation of microdomains of lipid rafts was also observed for PAMAM G7, G8 and G9 interacting with a bilayer mixture of 1,2-dimyristoyl-*sn-glycero*-3-phosphoglycerol (DMPG) with DMPC (3:7 ratio) lipids using coarse-grained simulations with the MARTINI force field. This simulation showed that the number of DMPG lipids near the dendrimer increased with the increase in dendrimer generation and a significant bending in the membrane was observed [146]. Overall these simulations are consistent with experimental data on dendrimers’ toxicity. Dendrimers, particularly high in generation and cationic, can cause deleterious effects by disturbing the membrane. The particular interest of performing molecular dynamics is not only to explain how these effects occur but also to prevent them by designing structures that do not favour these types of interactions. In particular, it would be of high interest to develop dendrimers that could efficiently encompass a mechanism of lysosome escape without the disruptive mechanism. This would be useful for a vast range of therapeutic molecules including gene therapy.

### 3.4. Modeling Dendrimers for Drug Delivery Applications

Combining the power of explanation of molecular mechanisms and construction of predictive models that can be applied to other kind of dendrimers gives a significant advantage to perform optimizations before even starting the synthesis of novel dendrimers. In terms of drug delivery applications, there are several factors where modeling and molecular dynamics can provide useful insights on how to optimize these carriers. Some of these factors include: (i) the stability of drug-dendrimer complexes; (ii) the strength of interactions that might compromise drug release; (iii) the availability of the targeting groups for interaction; (iv) the exposure of labile molecules to the solvent; (v) elucidate which forces govern the dendrimer-drug interaction and if those can be changed and finally; (vi) if can the dendrimer withhold a significant number of molecules to make it attractive to drug delivery. The detailed analysis of these factors is also crucial to understand how these dendrimers will perform* in vivo*.

**Figure 9:**
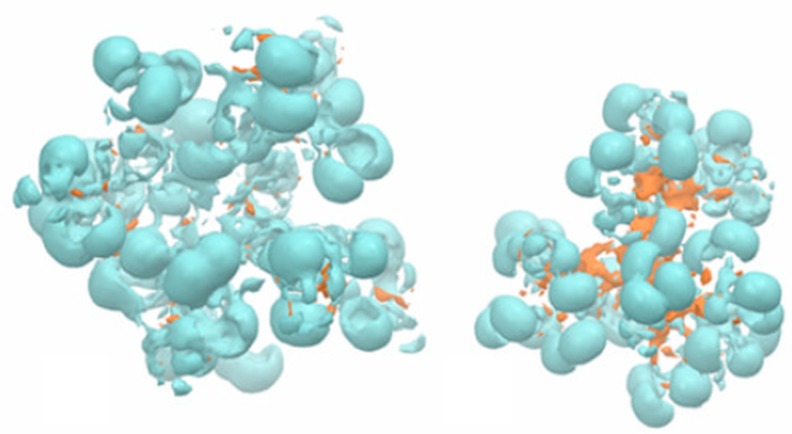
PAMAM dendrimers’ hydrophilic surface (blue) and hydrophobic core (orange). Adapted from [45].

In the field of drug delivery, dendrimers could be designed to a vast range of drug molecules in order to solve problems such as solubility, drug release and targeting. Essentially, drugs can be incorporated into the dendrimer either covalently (e.g., prodrug) or non-covalently (surface or internal cavities). As observed in the classical structural behaviour of PAMAM dendrimers, the hydrophobicity of the interior (see Figure 9) can be modulated through pH and salt variations. These variations can be used to ascertain the conditions for encapsulation that will be different from the medium in which they are going to be released.

A classic example is the delivery of nonsteroidal anti-inflammatory drugs (NSAIDs). The particular interest in these drugs lies in their potential for encapsulation and controlled release. This would be technological beneficial as a prolonged analgesic effect could be achieved without increasing side effects such as ulceration. In particular, ibuprofen is a common model drug to test new drug delivery systems due to its small size and being a class II drug (high permeability but low solubility) and hence the availability is limited by their solvation rate. Experimentally, it was shown that PAMAM dendrimers were able to solubilize ibuprofen [147,148], it was shown that this effect was dependent on the pH and generation of the PAMAM dendrimers.

To probe how the interaction occurs, a PAMAM G3 was simulated at different pH with ibuprofen [54]. Using all-atom MD simulations, with the AMBER FF, the drug molecules were placed in the proximity of the dendrimer. The analysis of atom distributions showed that ibuprofen could penetrate more to the dendrimer core than water. Unlike at basic and neutral pH, at acidic pH the ibuprofen is located homogenously throughout the dendrimers’ surface but cluster formation was observed. The measurement of the average distance between the ibuprofen and the PAMAM center of mass as a function of time also revealed a constant value at neutral and basic pH values, meaning the complexes formed are stable. At neutral pH conditions, hydrogen bonds are established between the dendrimer and the ibuprofen and this complex is mainly formed at the surface. On the contrary, at acidic pH, the ibuprofen diffuses away, this is seen by the increased values of mean distance [54]. This was consistent with the experimental results where the solubility of PAMAM dendrimers decreases under acidic conditions [147].

In another study, the calculation of dendrimer-ibuprofen complexes with incorporation of different amounts of drugs was evaluated using MM2 calculations. From these calculations it was observed that incorporating more than 16 ibuprofen molecules inside the dendrimer was energetically unfavorable [107], which is a similar value to the one found experimentally (14 ibuprofen molecules) for a PAMAM G3 [148].

Similarly, PPI G5 were simulated with Bengal rose with the DREIDING force field and these simulations revealed that depending on the drug:dendrimer ratio, the number of molecules found in the interior would vary as measured by distance from the center of mass of each molecule, in clear agreement with experimental data [84].

This kind of study can also be applied to other drugs of the same class, to study, which one showed better binding affinity to the dendrimer. Thus a predictive model can be built of the drug more suitable for the carrier. In this case the design is based on the selection of the drug rather than the optimization of the carrier itself. In order to have a more accurate calculation of the binding energies of four different NSAIDs drugs, semi-empirical methods (PM6-DH+) were used [149]. However, this approach is limited by the size of the dendrimer to be included in the calculations. To overcome this limitation, several branches of the dendrimer were taken separately from structures originated from MD simulations in NAMD on PAMAM G4 with CHARMM FF. The conformational pairs between branches and drug were then generated by a Monte Carlo method and the energetics calculated using semi-empirical methods (PM6-DH+). The energy values obtained were directly correlated with the affinity degree found experimentally (naproxen > ketoprofen > ibuprofen > diflunisal) [149].

In a similar study, but applied to anti-cancer therapy, MD simulations were carried out to assess the molecular interactions between a series of 24 cytotoxic drugs and a G4 PAMAM dendrimer. The results indicate that the majority of drugs show high thermodynamic stability. The complete set of drugs showed to effectively interact with the dendrimer in an exothermic fashion, with bleomycin, orlistat and porfimer being the ones that most strongly interact with the PAMAM dendrimer [150].

These are encouraging results has one can predict from a pool of interesting drugs which ones will fit best into the features of a given dendrimer. This kind of approach has been tested on PAMAM G5 dendrimers with different drugs (salicylic acid, L-alanine, phenylbytazone and primidone) [98]. These drugs were docked to the dendrimer via AutoDock Vina and then the best scoring conformations were selected for MD simulations using AMBER with explicit water solvent. Umbrella samplings were performed between the center of mass of the dendrimer and the drugs. When plotting the potential mean force (PMF) among all drugs studied l-alanine showed lower free energy (better ability to be released) followed by salicylic acid, primidone and phenylbutazone. However, taking into account the experimental data, although l-alanine and salicylic acid had a lower free energy (semi-empirical calculations) they were difficult to encapsulate in the dendrimer due to absence of nonpolar groups. This is due to less van der Waals contributions and the hydrogen bonding that did not contribute significantly to the free energy barrier. The PMF was also found to be less when the drugs are bound to the non-protonated dendrimer. The authors suggest that drugs should be encapsulated at higher pH and once in physiological pH they will be more tightly bond making the release controlled [97].

Proposed alteration of pH to modulate the interaction, encapsulation and release of drugs was observed from MD/MM simulations of PAMAM G5 and folic acid-terminated PAMAM G5 with tramadol and morphine [96] as well as PPI dendrimers G5 with Famotidine and Indomethacin [44] where the affinity of these drugs decreased with the decrease in pH. Similarly, PAMAM G4 equilibrated using MM+ FF were docked with resveratrol, genistein and curcumin [55]. The MD simulations revealed that the free energy binding followed genistein > curcumin > resveratrol which was in different binding constants determined experimentally which followed the order of curcumin > genistein > resveratrol. The difference between the calculation of energy of binding and the binding constants found experimentally was attributed to the difficulty of the drug to access the interior of the dendrimer.

A way of accounting for this kind of behavior could be by using adaptive biasing force methods. This kind of approach was recently used to study the association of nicotinic acid with a PAMAM G3 at different pH (pH 3 and pH 6) [95]. Using a biasing force method the drugs were constrained in the z-axis to make sure that the drug moved along the selected sample direction but were allowed to move freely through the x and y axis. The energy profiles showed that the nicotinic acid interacted better with G3 at higher pH with an energy difference of −1 to −2.5 kcal in comparison to 3-pyridiniumcarboxylate and that this interaction was more favorable at the surface than in the interior [95]. Since nicotinic acid is also a poorly soluble drug and needs to be delivered to the interior of the cells, determining at which conditions it can be better encapsulated in the dendrimer and its location served as a guide future optimization of the carrier.

A different kind of approach was used to study the stability of complex formation between poly(L-lysine) G6 dendrimers with the anticancer drug doxorubicin to evaluate the potential as a drug delivery system. The drug-dendrimer complex was found to be favorable at 300 K but dissociated upon heating up to 1000 K. However, once the system was cooled again it reassembles again showing the favorable interaction between these two molecules and hence favorable for controlled release [83].

One of the most promising areas for the use of dendrimers as drug delivery systems is gene delivery. Dendrimers have long been recognized as potential carriers to nucleic acids due to their highly expressed positive charge, which allows them to form polyelectrolyte complexes (also known as dendriplexes). Both experimental and all-atom MD simulations have shown that nucleic acids have the ability to wrap around the dendrimer in a process that depends both on size and charge ratio. In this particular application MD simulations can probe with substantial detail whether a dendrimer will be a suitable carrier. The mechanistic details that can be probed, including how strongly the nucleic acid binds to the dendrimer, which will impact on the release. If the binding is too strong, the release could be jeopardized, however, if the interaction is not as strong, the nucleic acid will be available to be cleaved in solution.

MD simulations were carried out in AMBER7 with AMBER95 FF for ssDNA and the DREIDING FF for the dendrimer, with different levels of protonation. The various dendrimers were docked to the major groove of an ssDNA. Further simulations were carried out in explicit water and counter ions were added to neutralize the system. The simulations showed that for G2 and G3 the charge ratio was not enough to complete wrap the ssDNA onto the dendrimers, as evidenced by the radius of gyration of the complexes. On the other hand the G4 dendrimer was large enough to neutralize the charges of the ssDNA and promoted the collapse of the latter into the surface. This led to the formation of a compact complex with significant penetration inside as measured by the radial density distribution. At neutral pH a higher degree of ssDNA penetration inside the dendrimer was observed. However, this may not constitute an advantageous phenomenon as it will prevent the release from the dendrimer and hence it is not useful as a gene delivery [49]. Similarly, protonated G3 and G4 PAMAM dendrimers with linked ssDNA were simulated using AMBER03 FF for ssDNA, GAFF for the linker and DREIDING FF for the dendrimer. Again, the ssDNA tended to loose helicity and wrap around the dendrimer with higher wrapping and DNA penetration in the case of G4 dendrimer [151].

Even when dsDNA (B-form) was simulated with G3 to G5 PAMAM dendrimers, a strong deformation of the DNA was observed [152]. Carrying MD simulations with AMBER03 FF in explicit water and added ions, the G5 expanded to try to cover the whole DNA while the DNA wrapped around the dendrimer as measured by the radius of gyration [152]. At the initial phase of the complex formation the dendrimer expands with increasing the contact between the dendrimer and DNA. Water molecules then suffer repulsion from the DNA backbone and DNA wraps on the dendrimer, forming a more stable complex. However, this phenomenon seems to be limited to the number of generation as it was shown by MD of PAMAM G7 dendrimers with siRNA, where the dendrimer behaved as a hard sphere with no variation in Rg after binding [99]. Higher charge ratio implies higher binding interaction [152]. Although the G3 is not enough to neutralize the DNA and a weak interaction occurs, this might be a better system as the release should be easier than in the case of a G4 or G5 [152].

In this regard, flexible triazine dendrimers of different generations and PEI polymer were simulated with DsiRNA in AMBER with parm99 FF to predict their efficacy [153]. Thermodynamically, dendrimers were found to be more stable than PEI with G2 being the most stable complex followed by G4 and G3. Furthermore, the charge neutralization of 1:1 complex predicted the stability of these complexes in solution as it was hypothesized that PEI only interacted partially with the DsiRNA. The authors suggested that the non-complexed part with both positive and negative charges promoted inter-particle interactions leading to aggregation. Finally* in vivo* studies showed that the G4 was more stable in comparison to the G2 (less excretion) but were significantly more uptake by the reticulo-endothelial system [153].

The sequence of the DNA also takes a role in the dendrimer-nucleic acid complex formation and thus the importance of using computational methods to predict this interaction. Using MD simulations with different strand composition it was found that the binding constant follows as polyG > polyC > polyA > polyT sequence as observed by the free energy calculations [49]. The flexibility or rigidity of dendrimers is another crucial point to form the polyelectrolyte complexes which was described to be due to the balance between the enthalpy and entropy of binding [98].

Dendrimers are also potential systems to be used as contrast agents since they can modulate the pharmacokinetic profiles and organ selection. Gadolinium-based triazine dendrimers with DOTA chelate groups was studied as contrast agent using MD simulations with AMBER FF. In this model, the G3 and G5 dendrimers had 24 and 96 chelates respectively [91]. The analysis of the radial distribution functions showed that the chelates were exposed to the solvent and available for chelation of Gd ions. In fact the high peaks in the RDF suggest that there is a reduced backfolding throughout the simulation time.

The multivalence of dendrimers is one of its upmost regarded advantages in which they can be design to perform multiple actions within the same structure. This is particularly useful in the application of imaging agents. In this regard, a PAMAM G5 linked to gold nanoparticles with randomly distributed targeting ligand (folic acid) and imaging agent (fluorescein isothiocyanate) were simulated with CVFF [93]. The MD simulation was particularly useful in identifying that the fluorescent probe was at a significant distance from the gold metal and therefore a low quenching was expected but also that the folic acid was extended outwards making it available to interact. This system is interesting as it is an “all-in-one package”. This dendrimer system could make selective targeting of the cancer, highlight where it is present and then laser hyperthermia could be induced on the gold nanoparticle.

### 3.5. Modeling Dendrimers as Therapeutic Agents

Dendrimers due to their inherent similarity to biological macromolecules such as proteins can interact with other biomolecules and illicit biological responses [48,52]. Their high number of terminal groups promotes multivalent non-specific contacts that are beneficial for biological interactions. Furthermore, specific modifications can be introduced at the surface to promote binding. Using simulations on PAMAM G0 modified with guanidinium it was found that these modifications promoted simultaneously and cooperatively interactions with the protein surface of α-chymotrypsinogen A by hydrogen and cation-π interactions with the aminoacids [73]. This cooperative interaction was found to promote higher binding compared to a single unit.

Dendrimers can be designed and modelled to specifically interact with the desired target. PAMAM G3.5 dendrimers were modelled to predict the availability of glucosamine moieties at the surface and their influence on biological activity. Experimentally, PAMAM G3.5 (with carboxylic groups) with an average of 8 surface glucosamine molecules were found to inhibit the TLR5-MD-2-LPS complex formation [154]. Frontier molecular orbital theory (FMOT) was applied on smaller dendrimer dimensions to predict the reactivity (gap difference between HOMO and LUMO) of the dendrimer towards subsequent addition of glucosamine monomers [90]. These studies found that solely based on electronic properties of the dendrimer a maximum of 12 molecules cold be coupled to the structure. However, upon addition of more glucosamine steric effects of hindering the terminal groups started to occur as evidenced by MD simulations. Together these effects accounted to an average of 4–8 as the most favorable energetically [90].

**Figure 10:**
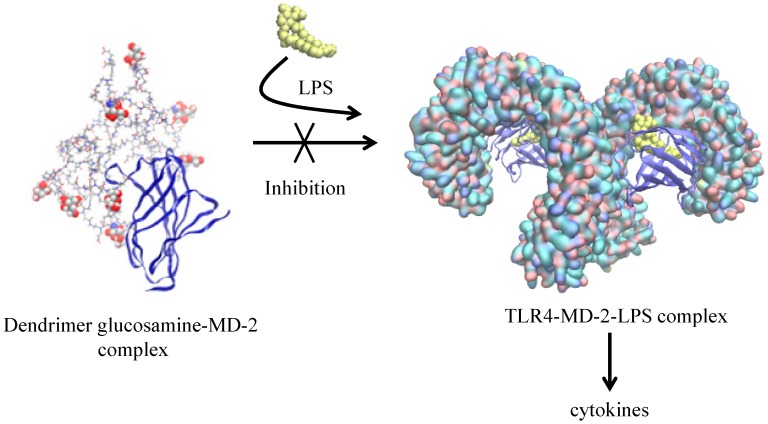
Docking between Dendrimer glucosamine and MD-2 protein and inhibition of TLR4-MD-2-LPS complex formation; adapted from [52].

These dendrimers were subsequently docked to MD-2 protein followed by a MD simulation. These results showed that the dendrimer could indeed act as an antagonist of the MD-2 receptor [52] (see Figure 10). Analysis of the trajectory showed that the PAMAM-glucosamine’s groups could cooperatively bind to the hydrophilic residues at the entrance of the hydrophobic pocket [52]. This action was enough to block the entrance of LPS into it. This effect was very important as it was shown to prevent the formation of the TLR4-MD-MD-2-LPS from initiating the cytokine cascade. The predictive knowledge of this dendrimer-glucosamine derivative to the receptor was used as the basis for further design of the dendrimer architecture.

Active and non-active PETIM [155] dendrimers and Triazine-PAMAM hybrids [45] were modelled on the same basic principles. The modified PETIM dendrimers showed similar flexibility and the net surface charge was found to resemble that of the PAMAM derivative. This dendrimer was particularly interesting as it was a lower molecular weight compared to the original PAMAM dendrimer (easier synthesis and purification as well). The results from the non-active forms of this dendrimer revealed that the surface hydrophobicity did not contributed to the interaction with the MD-2 receptor. Similarly in the case of the Triazine-PAMAM hybrids [45] it was found that the best architecture to promote antagonist effect was that of partially glycosylated G2 Triazine G1.5 PAMAM hybrid dendrimers. This dendrimer resembled the active PAMAM G3.5 glucosamine in key points such as polar surface area and globular shape (contrary to pure Triazine). These features facilitate the electrostatic interactions with the target and may justify its activity.

Another interesting application of dendrimers in the biomedical field is in the area of vaccination. Here the core base dendrimer can be used to attachment of multiple groups depending on the type of immunization required. Using a database of *Plasmodium falciparum* epitopes, several epitopes were selected and attached to the dendrimer [156]. These systems were simulated using CHARMM FF and explicit water, and the energetics of the different structures was calculated. Although not many details are given, this is an interesting application of MD simulations as one could estimate the availability of the epitopes to the solvent. It can also be used to see the effect of adding other groups such as recognition groups or even fluorophores to track the intracellular path of these dendrimers.

Even though dendrimers can be designed to specifically interact with certain proteins, while circulating in the plasma they will contact other proteins that can cause significant alteration of the pharmacokinetic profile. A common test is to study the binding affinity to albumin, the most abundant human plasma protein. In particular, dendrimers with their high number of terminal groups can promote unspecific binding and therefore bind to plasma proteins. Molecular modeling offers a potential test to evaluate and modulate these interactions. Using the previously described DREIDING FF that predicted the behavior of PAMAM at different pH, PAMAM dendrimers were simulated with human serum albumin to estimate the contacts between the two. It was suggested that the size and surface of terminal groups were crucial for this interactions. The interaction between dendrimer-HSA was due to electrostatic interactions between the charged groups as well as hydrogen and hydrophobic interactions. This interaction resulted in a backfolding conformation of PAMAM dendrimers with the inner groups participating in this interaction [47]. SASA measurements were calculated to estimate relevant contact areas with different probe radii size. From both simulations and the NMR data it was suggested that PAMAM form weak complexes with HAS [47].

## 4. Challenges and Perspectives

Currently, there are over 100,000 entries in the protein databank (PDB) with experimentally determined structures of various biologically important molecules. In contrast there are only nine entries that have structures of dendrimer components reported. This is a clear indication that dendrimers have intrinsic properties that prevent experimental elucidation of their 3D structures and their interactions with components of biological systems. Computational modeling and molecular dynamics have been valuable tools for design and optimization of drug candidates and polymers in the past. Therefore, there is a growing interest in the opportunities to apply computational chemistry tools to study dendrimers. Molecular modeling when effectively applied on dendrimers will offer a means to study conformation and many features of the dynamic behavior of dendrimers on a molecular level that are difficult to probe experimentally. These approaches will also allow the elucidation of some of the key interactions of functionalized dendrimers with therapeutic molecules and biological systems such as proteins and lipid membranes.

There are currently two main challenges in the modeling of dendrimers. The first is to obtain the initial three dimensional coordinates of the dendrimer to be used in further computational studies. This task can be performed manually by sketching the dendrimer with common chemical drawing tools with relatively ease for small generation dendrimer. However with more interesting and larger generation dendrimer, this approach is prone to errors. Furthermore there are no guidelines on how the initial structure should be assembled although it can be easily imagined that a fully stretched starting structure should be obtained to prevent dendrimer being stuck in a local minimum of the initial structures [76,105].

To the best of our knowledge there are only two tools that attempt to generate the starting three dimensional coordinates of a dendrimer of interest. Dendrimer Builder Toolkit [70] is a graphical user interface written in PERL that interfaces with AMBERTOOLS to build dendrimers. The other tool, also written in Perl, is called Silico [69], which has a module named Starmaker that generates *mol2 file of dendrimers by building them layer by layer. Our group is developing a an alternative way of building dendrimers or other hyperbranched polymers using XPLOR [71] through the definition of a sequence of monomers and the way they are connected to each other through patch references. The structures generated using this approach can be converted to file formats with more general force fields from XPLOR topology and parameter files. We are currently working on a python GUI to automate the process of assembly.

Another major issue with computationally simulating dendrimer structures is that there is no dedicated force field for this kind of macromolecule. This is probably due to the wide range of chemical bonds used to make dendrimers and that there are only few crystal structures of dendrimers. As a result, studies often rely on force fields developed for other macromolecules such as proteins with optimization of specific parameters or the use of more general force fields and these have been shown to be generally appropriate to predict and clarify experimental data.

Developing more general atom types in force fields will allow the automated generation of dendrimer structures, which will help to determine structure-property correlations needed to gain insight into their conformational behavior. Furthermore these generated dendrimer structures could be used in docking experiments. These types of study allow a dendrimer to be used as targets for small molecules or as ligand for biopolymers (protein or DNA structures). The docking solutions can be used in MD simulations to explore detailed intermolecular interactions. Docking studies also allow for selection of optimal dendrimer architecture for synthesis and further optimization.

Despite the current challenges with the lack of dedicated force fields and tools to easily represent dendrimer structures, interest in dendrimers for biomedical applications remains high together with the increase use of computational methods to predict or validate experimental data. While most of the simulations have been conducted for PAMAM dendrimers, and to a lesser extent for poly(L-lysine) and triazine dendrimers, this is understandable by the availability of these dendrimers. It is hoped that sufficient MD data about other types of dendrimers will become available. Continued computation studies of dendrimer will yield new methods of analysis of their dynamic structural properties, which will be essential to their design and optimization. Finally, the expected increase in availability of computational power will allow the extension simulation times to probe more complex interactions, such as dendrimer-membrane interaction. Our hope is that the molecular modeling will become more widely used for dendrimers and its usefulness will compare to that which currently exists for proteins.
